# *FGFR2* amplification in colorectal adenocarcinoma

**DOI:** 10.1101/mcs.a001495

**Published:** 2017-11

**Authors:** Jamal H. Carter, Catherine E. Cottrell, Samantha N. McNulty, Katinka A. Vigh-Conrad, Stephen Lamp, Jonathan W. Heusel, Eric J. Duncavage

**Affiliations:** 1 Department of Transfusion Medicine, Clinical Center, National Institutes of Health, Bethesda, Maryland 20892, USA;; 2 Department of Pathology and Immunology, Washington University in St. Louis, Missouri 63130, USA;; 3 Department of Genetics, Washington University in St. Louis, Missouri 63130, USA

**Keywords:** colon cancer, neoplasm of the gastrointestinal tract

## Abstract

*FGFR2* is recurrently amplified in 5% of gastric cancers and 1%–4% of breast cancers; however, this molecular alteration has never been reported in a primary colorectal cancer specimen. Preclinical studies indicate that several FGFR tyrosine-kinase inhibitors (TKIs), such as AZD4547, have in vitro activity against the *FGFR2*-amplified colorectal cell line, NCI-H716. The efficacy of these inhibitors is currently under investigation in clinical trials for breast and gastric cancer. Thus, better characterizing colorectal tumors for *FGFR2* amplification could identify a subset of patients who may benefit from FGFR TKI therapies. Here, we describe a novel *FGFR2* amplification identified by clinical next-generation sequencing in a primary colorectal cancer. Further characterization of the tumor by immunohistochemistry showed neuroendocrine differentiation, similar to the reported properties of the NCI-H716 cell line. These findings demonstrate that the spectrum of potentially clinically actionable mutations detected by targeted clinical sequencing panels is not limited to only single-nucleotide polymorphisms and insertions/deletions but also to copy-number alterations.

## INTRODUCTION

Colorectal cancer (CRC) is a leading cause of mortality and morbidity and constitutes the third most common malignancy diagnosed in both men and women (Siegel et al. 2016). CRC tumors are generally divided into two broad, but biologically distinct, molecular subtypes: tumors characterized by microsatellite instability (MSI) and tumors characterized by chromosomal instability (CI). MSI occurs when mismatch repair (MMR) pathways become inactivated, either through methylation of the *MLH1* promoter (typically in a CpG island methylator phenotype background) or through mutational inactivation of *MLH1* and/or other MMR pathway genes. MSI tumors constitute ∼15% of sporadic CRC cases. They typically present in the right hemicolon and are associated with *BRAF* V600E mutations (Cancer Genome Atlas 2012). CI tumors constitute ∼85% of sporadic CRC cases. This heterogeneous group is characterized by mutations in *APC, KRAS*, and *TP53*, as well as frequent, recurrent somatic copy-number alterations (SCNAs) (Cancer Genome Atlas 2012; Wang et al. 2015).

Although many recurrent mutations have been identified in CRC, only a few have been validated as predictive biomarkers. Anti-EGFR antibody therapy (e.g., panitumumab and cetuximab) has proven effective in metastatic tumors with wild-type MAPK pathway genes (Amado et al. 2008), but mutations in tumors with *KRAS*, *NRAS*, and/or *BRAF* confer decreased sensitivity to these drugs (National Comprehensive Cancer Network 2016). Likewise, the clinical importance of most of the reported SCNAs is uncertain (Wang et al. 2015), but rarely a clinically actionable discovery is made. In a case report of a patient with CRC with amplification and overexpression of *ERBB2*, a dramatic radiographic response was observed upon treatment with trastuzumab (Sorscher 2011), similar to the response observed in *ERBB2*-amplified metastatic breast cancer (Vogel et al. 2002).

Here, we present the multiplatform, pathologic characterization of a case of CRC submitted for routine clinical analysis. This sample was subjected to next-generation sequencing (NGS) following hybrid capture–based enrichment of a 425-kb target space. Although this assay was intended to detect single-nucleotide variants and small insertions and deletions (indels) in cancer relevant genes, a read depth (>5000×, reference mean 1135×) suggestive of a focal amplification was noted at the *FGFR2* locus, a SCNA that is considered medically actionable in other cancer types. Bioinformatic software designed to predict SCNA from tumor samples confirmed the *FGFR2* amplification from sequencing data, and findings were verified by chromosomal microarray and fluorescence in situ hybridization (FISH). This is the first report of a *FGFR2* amplification in a non-cell-line-derived, clinical CRC specimen obtained during the course of routine pathologic examination. This case highlights the utility of SCNA prediction from clinical NGS data, as copy-number variations can be detected in the absence of predefined expectations.

## RESULTS

### Clinical Presentation

**Figure 1.**
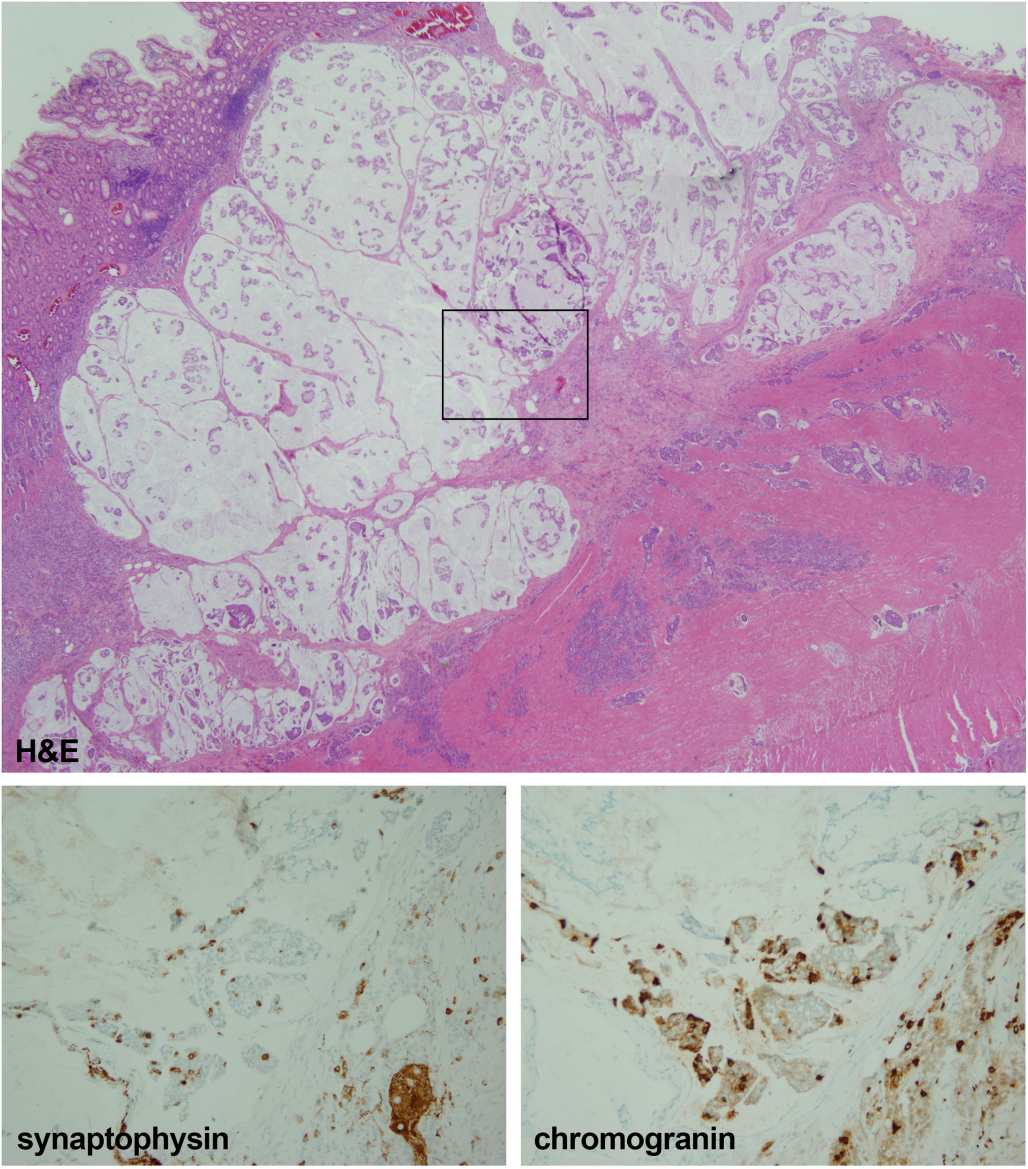
Neuroendocrine marker immunostains. (*Top*) H&E, 2×. Poorly differentiated, signet-ring adenocarcinoma showing abundant mucin in this section. (*Bottom left*) Synaptophysin, 20×. Focal expression present in the malignant cells. (*Bottom right*) Chromogranin, 20×. Malignant glands showing patchy expression.

**Figure 2.**
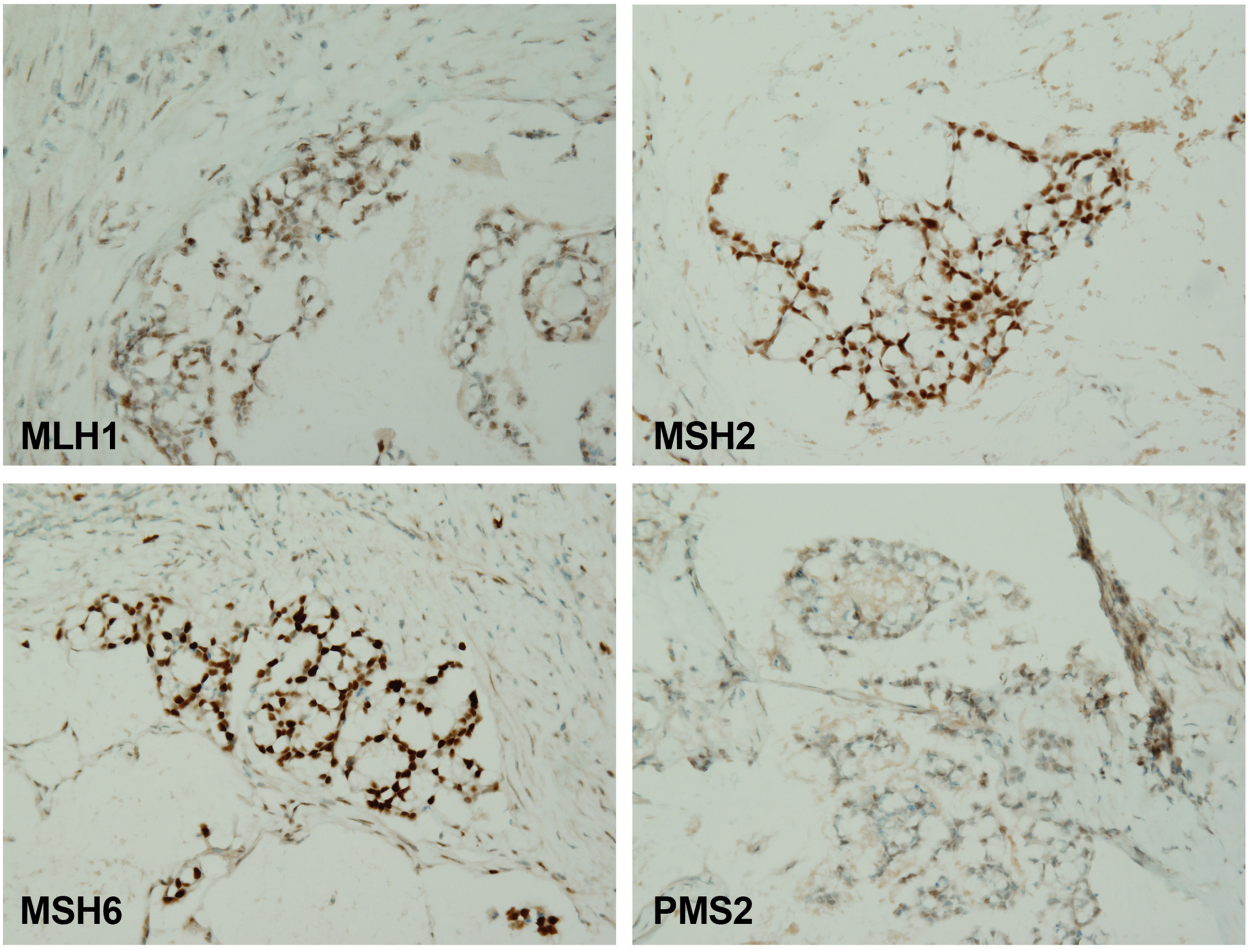
MMR immunostains showing retained expression of all four MMR markers. (*Top left*) MLH1, 20×. (*Top right*) MSH2, 20×. (*Bottom left*) MSH6, 20×. (*Bottom right*) PMS2, 20×.

A 56-yr-old female with a questionable history of ulcerative colitis underwent a total abdominal colectomy for a 6-cm, poorly differentiated, signet-ring adenocarcinoma in the sigmoid colon (Fig. 1, top inset). The specimen revealed transmural and serosal involvement of the perirectal and pericolic soft tissue by the tumor, as well as widespread lymphovascular space invasion and metastatic involvement of 14 of 18 pericolonic lymph nodes. Altogether, this constituted stage IIIC disease. MMR markers MLH1, PMS2, MSH2, and MSH6 showed retained expression by immunohistochemistry (IHC) (Fig. 2).

Adjuvant chemotherapy with a standard first-line regimen (FOLFOX) was initiated but stopped after eight cycles because of side effects. Further imaging was performed to assess the disease status. This revealed bulky retroperitoneal and portahepatic lymphadenopathy, multiple metastatic, cardiophrenic lymph nodes, and multiple hepatic lesions. A fine needle aspiration of a liver lesion confirmed metastasis, indicating stage IV disease; however, there was insufficient tissue for further molecular studies. An alternative regimen (FOLFIRI) was then initiated, and tissue from the patient's primary colon cancer resection specimen was submitted to Genomics and Pathology Services at Washington University in Saint Louis (GPS@WUSTL) for mutational profiling. The GPS panel (described below) is a next-generation sequencing–based panel that targets 48 common cancer genes and is performed in a certified clinical environment, permitting return of patient results and third-party billing.

### Genomic Analysis, Microarray, and FISH

A single reportable mutation, *TP53* p.D281E (Chr17:g.7577095G>T; NM_000546:c.843C>A), was identified among the 48 genes analyzed on the GPS@WUSTL solid tumor panel (Table 1). According to the COSMIC (Forbes et al. 2015) and IARC *TP53* mutation (Petitjean et al. 2007) databases, the p.D281E variant in *TP53* has been described as a deleterious somatic mutation in various carcinomas, although it has not been specifically previously reported in CRC. Functional data from promoter response element transactivation experiments indicate that this variant results in loss of protein function (Jordan et al. 2010).

**Table 1.** Detected somatic variants

Chr	Location	Class	Gene	Allele change	RefSeq ID	cDNA pos	AA change	Mutation	dbSNP	SIFT score	PolyPhen-2 pred	Mutation type	Cancer gene	No. of reads ref/alt	Genotype
Chr17	7577095	SNV	TP53	G>T	NM_000546	NM_000546:c.843C>A	NP_000537:p.D281E	missense	N/A	0.01	0.973	somatic	Y	423/405	Het

AA, amino acid; dbSNP, Database for Short Genetic Variations; SIFT, Sorting Intolerant from Tolerant; PolyPhen-2, Polymorphism Phenotyping v2; SNV, single-nucleotide variant.

**Figure 3.**
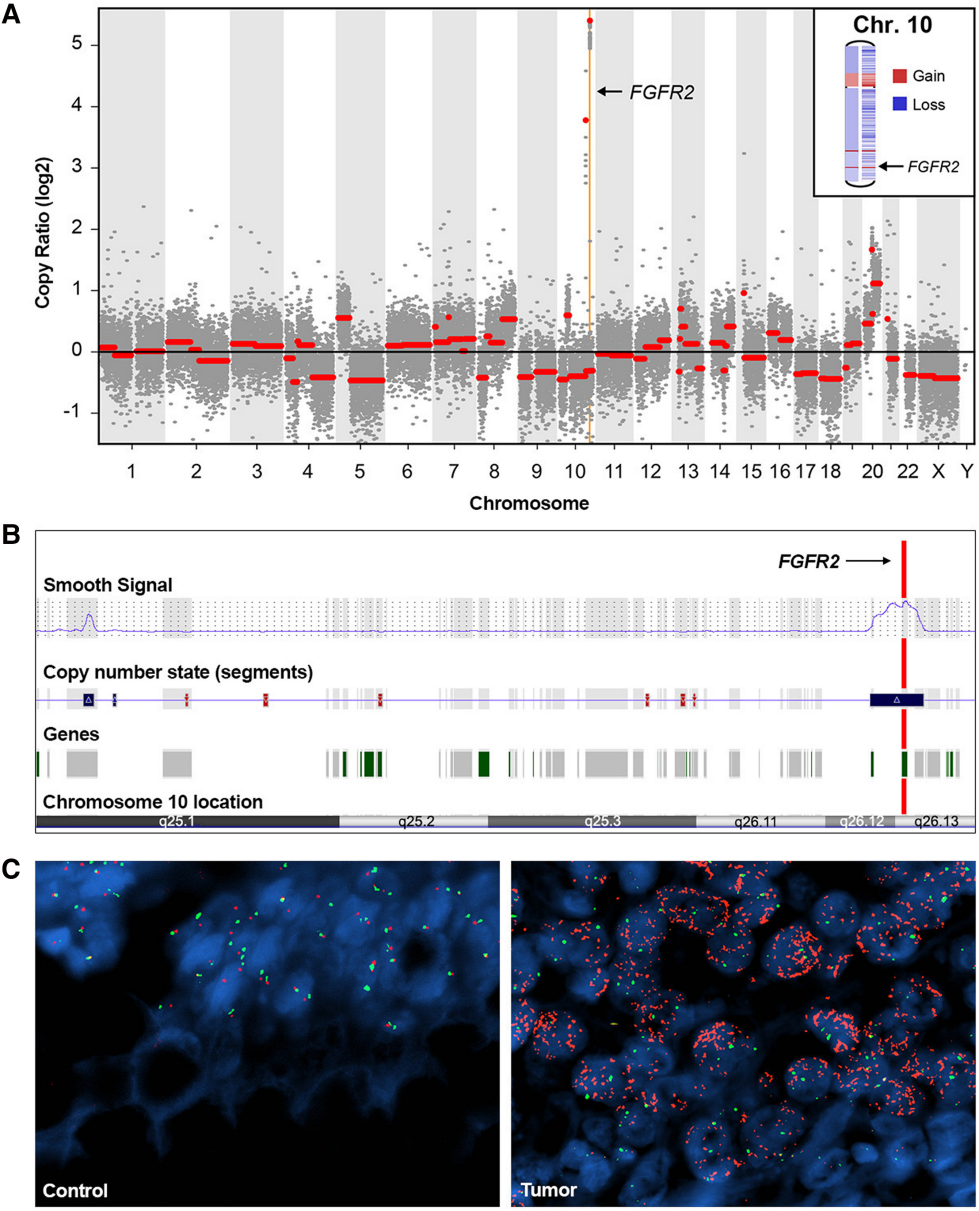
Multimodal analysis of *FGFR2* in the tumor. (*A*) Targeted NGS-based virtual karyotype (CNVkit) showing background aneuploidy and an amplification event involving a focal region of Chromosome 10 (Chr10:122737302–123454446) that includes *FGFR2*. (*B*) Cytoscan microarray output from the tumor tissue revealing amplification of the region involving *FGFR2*. (*C*) Fluorescence in situ hybridization of tumor-involved (Tumor) and tumor-uninvolved (Control) colonic tissue from the patient's colectomy. *FGFR2* probe (RP11–62L18, red) to Chromosome 10 enumeration probe (CEP 10, green) ratio showing a high copy-number ratio of 24.2 in the tumor (right) versus 1.92 in the control (*left*).

Standard quality control metrics indicated significant depth of coverage in the region of the *FGFR2* gene. To further explore the possibility of copy-number variation (CNV), genome-wide copy-number information was extracted from the targeted NGS data using two somatic copy-number aberration detection tools: CopywriteR (Kuilman et al. 2015) and CNVkit (Talevich et al. 2014, 2016). Both tools revealed a focal copy number amplification involving the *FGFR2* locus in a background of chromosomal aneuploidy (Fig. 3A,B). Consistent with NGS read depths, microarray data confirmed the presence of a focal, somatic *FGFR2* amplification (Fig. 3B). The 1.08-Mb amplified region encompassed the entirety of the *FGFR2* gene, along with the 3′ genic regions of *WDR11* and *ATE1* (hg19 Chr 10:122,608,791-123,684,530). FISH also revealed a striking amplification of *FGFR2* in tumor-involved tissue (Fig. 3C). Among 100 analyzed nuclei in the tumor tissue, the average RP11-62L18 probe signal corresponding to *FGFR2* was 63.91, whereas the average chromosome enumeration probe (CEP) 10 control signal was 2.64, yielding a ratio of 24.2. Analysis of the patient's normal colonic tissue demonstrated an average RP11-62L18 (*FGFR2*) signal number of 1.92 and average CEP 10 signal number of 1.87, yielding a ratio of 1.02. *FGFR2* gene amplification has only recently been documented in CRC, where it was identified in the NCI-H716 colon cancer cell line (Mathur et al. 2014).

### Protein Expression Analysis with IHC

The NCI-H716 CRC cell line harboring the *FGFR2* amplification has features of endocrine differentiation, which is unusual for CRC. IHC studies indicated that NCI-H716 shows expression of chromogranin A, mucin, glucagon-like peptide-1 (GLP-1), and receptors for gastrin, somatostatin, and serotonin (de Bruïne et al. 1992; Reimer et al. 2001). We followed up on this observation, assessing the extent of neuroendocrine differentiation in the patient's tumor by assessing the expression of synaptophysin and chromogranin A using IHC assays commonly implemented in the anatomic pathology laboratory. Patchy expression of chromogranin and focal expression of synaptophysin was noted in the malignant cells of the tumor specimen, comprising ∼10% and <5% of the total tumor cellularity, respectively (Fig. 1).

## DISCUSSION

NGS-based profiling of the tumor genome identified a single nonsynonymous mutation among the many genes noted for recurrent mutations in CRC (e.g., *KRAS, NRAS, BRAF, PIK3CA*) and a striking focal amplification of the *FGFR2* gene. *FGFR2* amplification is thought to occur in 5% of gastric cancers and 1%–4% of breast cancers (Heiskanen et al. 2001; Turner et al. 2010; Jung et al. 2012). In gastric cancer, *FGFR2* amplification is associated with poorer overall survival (Su et al. 2014; Shoji et al. 2015). Preclinical, in vitro, and in vivo studies suggest that breast and gastric cancer cell lines with *FGFR2* amplifications may be sensitive to FGFR inhibitors (Jain and Turner 2012; Xie et al. 2013); clinical trials are ongoing.

The only report of *FGFR2* amplification in CRC was from the NCI-H716 colon cancer cell line (Mathur et al. 2014). Through a series of functional assays, Mathur et al.’s (2014) study demonstrated that NCI-H716 cells are dependent on *FGFR2* amplification and overexpression for survival and proliferation, and they appear to be sensitive to FGFR inhibitors (Mathur et al. 2014; Medico et al. 2015). To ascertain the frequency of this molecular event and the resultant FGFR2 protein overexpression, Mathur et al. performed FGFR2 IHC on a series of primary and lymph node–metastatic CRC samples in a tissue microarray. Although they identified FGFR2 overexpression of five samples (1.5% of >300 tested from various body sites), FISH was negative for *FGFR2* amplification in all samples tested. Thus, this is the first report to identify *FGFR2* amplification in a CRC tumor sample obtained directly from a patient's primary tumor and the first non-cell-line-derived, clinical case harboring the aberration.

Although not reported as part of the clinical panel, analysis of the raw sequencing data by CopywriteR and CNVkit also demonstrated an amplification of the *ASXL1* gene (Supplemental Table 1); the *ASXL1* gene and 51 other genes were targeted by the clinical sequencing panel but were not analyzed or reported as the part of the solid tumor gene panel. The *ASXL1* gene is involved in chromatin modification and is mutated in up to 20% of myelodsyplastic syndrome cases and a smaller percentage of acute myeloid leukemias (Cancer Genome Atlas Research et al. 2013; Haferlach et al. 2014). In hematologic malignancies *ASXL1* mutations tend to be small insertions or deletions that result in frameshifts. However, *ASXL1* amplifications appear to be reasonably common in solid tumors and are present in >20% of uterine carcinosarcomas and ∼10% of colorectal cancers, according to TCGA data (Cancer Genome Atlas 2012; Zhao et al. 2016). *ASXL1* amplifications have no known prognostic significance in solid tumors and may be part of larger genomic events that span several genes on Chromosome 20.

The NCI-H716 cell line has been demonstrated to express chromogranin A, mucin, GLP-1, and receptors for gastrin, somatostatin, and serotonin (de Bruïne et al. 1992; Reimer et al. 2001). We followed up on this potential insight by assessing the extent of neuroendocrine differentiation of our case by utilizing the standard, routine IHC stains commonly used in the anatomic pathology laboratory for this purpose: synaptophysin and chromogranin A. We found the patient's tumor had focal to patchy staining of both markers, indicating some partial neuroendocrine differentiation, similar to the NCI-H716 cell line. However, it has been documented that up to 40% of CRCs may have detectable neuroendocrine marker expression by IHC (Bosman and World Health Organization 2010). Furthermore, according to the World Health Organization classification scheme for digestive tumors, a CRC specimen must show 30% of an adenocarcinoma and a neuroendocrine component, each, to qualify for classification as a “mixed adenoneuroendocrine carcinomas (MANEC)” (International Classification of Diseases, ICD10: C18–C20) (Bosman and World Health Organization 2010). Our case fell short of these criteria. Nevertheless, this association possibly suggests an interesting association that may warrant further inquiry. The spectrum of molecular abnormalities present in MANECs is a poorly studied area, with only case reports and small case series in the literature that primarily survey small genomic changes, such as single-nucleotide variants and indels of <10 bp (Scardoni et al. 2014; Vanacker et al. 2014). In one study that examined six cases of MANEC by targeted NGS panel, *TP53* mutations were the most frequently identified molecular abnormality (Scardoni et al. 2014). To the best of our knowledge, no study has comprehensively surveyed the SCNAs present in MANECs or CRCs with neuroendocrine differentiation, precluding the comparison of our case to the frequency of *FGFR2* amplification in neuroendocrine-expressing CRC based on the known, extant literature.

The striking signet-ring morphology of the tumor cells in this case, the lack of an identifiable adenomatous mucosal component, and the neuroendocrine differentiation are also very reminiscent of goblet cell carcinoids (GCCs) (International Classification of Diseases, ICD10: C23–C24). However, GCCs are anatomically located within the vermiform appendix, almost exclusively (Roy and Chetty 2010). Similar to the scenario with MANECs, no comprehensive SCNA study exists in the literature for GCCs, likely as a consequence of their rarity. Interestingly, the NCI-H716 index case is derived from the cecum, which is anatomically proximal to the appendix. Although NCI-H716 was formally diagnosed as a colorectal adenocarcinoma, and GCC had been described at least a decade prior to the development of the NCI-H716 cell line, GCC was not a widely recognized diagnostic entity at the time (Park et al. 1987; Pahlavan and Kanthan 2005; Gagnon and Brubaker 2016). Similar to the scenario with MANECs, no comprehensive SCNA study exists in the literature for GCCs. However, loss of heterozygosity was frequently identified in the 16 cases of GCCs included in a study that used targeted dinucleotide microsatellite markers for 11q, 16q, and 18q, a pattern associated with midgut carcinoids (Stancu et al. 2003). Chromosome 11 is present in a single copy in the NCI-H716 cell line, but Chromosomes 16 and 18 are present at normal levels (Atcc 2016). None of these cytogenomic abnormalities were identified in our case.

Finally, this case report demonstrates that potentially actionable copy-number alterations can be detected from NGS data generated from small, targeted, clinical NGS panels. Although only a minority of clinical laboratories currently report SCNAs as part validated clinical testing, evidence of large amplifications, such as this *FGFR2* amplification, can be suggested by a review of the coverage-based QC metrics at case sign-out. In the absence of a formally validated SCNA detection pipeline, clinical laboratories may opt to detect copy gains by simply imposing an upper boundary for gene level coverage (e.g., 5000×, given a mean reference coverage depth of 1132×). Going forward it has become increasingly clear that SCNA detection will become an important part of clinical molecular genetic testing.

In conclusion, the genomic, histologic, and IHC characterization of a clinical case of primary CRC with *FGFR2* amplification unveiled phenotypic and genotypic similarities with the index cell line case for the CRC cell line NCI-H716. Currently, it remains unclear how frequent *FGFR2* amplifications are in patients with CRC, much less in CRC with neuroendocrine differentiation or GCC morphology. The insight contributed by this report will be helpful in identifying the CRC patient cohort that can potentially benefit from FGFR tyrosine-kinase inhibitor therapy and in further establishing and clarifying the possible association between neuroendocrine differentiation, mucinous signet-ring morphology, and *FGFR2* amplification. Moreover, these findings demonstrate the utility of routine, clinical NGS panels in uncovering novel, potentially actionable, somatic mutations in cancer.

## METHODS

### Next-Generation Sequencing and Data Analysis

Genomic DNA was isolated from a formalin-fixed paraffin-embedded (FFPE) tissue block originating from the primary colon resection specimen using the QIAamp DNeasy Blood and Tissue Kit (QIAGEN), and sample quality and quantity was assessed by Qubit (Thermo Fisher Scientific) and Nanodrop (Thermo Fisher Scientific). Seven hundred and fifty nanograms of DNA was sheared in the Covaris S220 series sonicator to an average fragment size of 140–230 bp, as measured by Bioanalyzer (Agilent Technologies). Fragmented DNA was end-repaired, A-tailed, and indexed using the KAPA Hyper Prep Kit (KAPA Biosystems). Adapter-ligated DNA was subjected to limited amplification prior to hybridization with custom cDNA capture probes (IDT). The total size of the target space was 425 kb, encompassing all coding exons of 99 cancer relevant genes, selected introns, and intergenic regions targeted for quality control. The hybridized product was washed, amplified with the KAPA amplification kit, and sequenced on an Illumina HiSeq 2500 to generate 2× 101-bp paired-end reads.

Analysis and interpretation was performed as previously described (Hagemann et al. 2014).

Reads were aligned to the human reference (UCSC build hg19 / NCBI build 37.2) using Novoalign (Novocraft Technologies). PCR duplicates were marked with Picard Tools (version 1.53, http://picard.sourceforge.net), and alignment files were converted to mpileup format using SAMtools (version 0.1.18-0.1.19; Li et al. 2009). Single-nucleotide variants (SNVs) with depth ≥50× and Fisher's exact test of strand bias ≤100 were detected using VarScan2 (version 2.3.6; Koboldt et al. 2009), and visualized in Integrative Genomics Viewer (version 2.0.16 or later; Robinson et al. 2011). Reports were generated using the Clinical Genomicist Workstation v2.1.1 (PierianDx). Single-nucleotide variants with global mean allele frequencies in the population of >1% were considered “known SNPs” and excluded from this report. Nonsynonymous SNVs that were not known polymorphisms were deemed clinically relevant and reportable. Quality and gene-level read depth metrics are available in Supplemental Tables S1 and S2.

### Copy-Number Analysis

Copy-number profiles were determined by CopywriteR (Kuilman et al. 2015) and CNVkit (Talevich et al. 2014, 2016) using the same alignment files employed in SNV calling. CopywriteR was run with window size set to 50 kb and no matched or normal reference. CNVkit's standard batch pipeline was run with default parameters. Fifteen FFPE tissue samples from patients without cancer and previously processed through the same analysis pipeline were included as pooled controls to facilitate the required normalization for CNVkit; to further confirm the absence of CNAs in these 15 control samples, all cases were tested by CopywriteR (which does not require normal controls) and showed no evidence of CNAs.

### Microarray

Copy-number alteration was assessed using the Affymetrix CytoScan HD array (Affymetrix) containing approximately 2.67 million markers including 1.9 million nonpolymorphic probes and nearly 750,000 probes capable of single-nucleotide polymorphism (SNP) detection. DNA was derived from two sections of the patient's colectomy specimen representing tumor and normal colonic mucosa. Beginning with 1 μg of input DNA, the specimens were enzymatically digested, adaptor-ligated, and amplified prior to hybridization on the array platform. Data were derived using an Affymetrix GeneChip Scanner 3000 7G and copy-number status was determined in comparison to an in silico FFPE-specific reference file (CytoScanHD_Array.na33.r2.FFPE.v3.REF_MODEL). Analysis was performed using Chromosome Analysis Suite v3.1.0.15 (Affymetrix).

### Fluorescence In Situ Hybridization

FISH was performed using the BAC clone, RP11-62L18 (Empire Genomics), mapping to the *FGFR2* gene and with CEP 10 (Empire Genomics) mapping to the centromeric region of Chromosome 10. FFPE tumor-involved and normal colonic tissue from the patient were sectioned at 5 microns and mounted on positively charged slides. Corresponding hematoxylin and eosin (H&E)-stained slides were reviewed by a pathologist to mark for areas of tumor and normal tissue and then transcribed to the unstained slides for FISH. Slides were deparaffinized with Citra Solv and dehydrated in 100% ethanol before specimen pretreatment using the Pretreatment kit II (Abbott Molecular) including Pretreatment solution (NaSCN) at 80°C, Protease (Pepsin 2500–3000 U/mg, lyophilized) in Protease buffer (0.2 N HCI) at 37°C. After an ethanol dehydration series (70%, 85%, and 100%), slides were air-dried, and 12 µL of probe mixture was applied. Coverslipped, sealed slides were then placed in a preheated 73°C slide moat (Boekel Scientific), allowing the patient DNA and the probe to denature for 5 min, followed by an overnight hybridization at 37°C. Posthybridization wash was performed using 2× SSC at 74 ± 1°C and 2× SSC at room temperature before counterstaining with DAPI (4′,6-diamindino-2-phenylindole). Using a BX61 fluorescent microscope (Olympus), the nuclei containing the RP11-62L18 (*FGFR2)* probe signals and the CEP 10 control signals were examined within the areas marked, with 100 nuclei used to generate probe counts. Signal patterns were documented using the Jai Progressive Scan camera and CytoVision Imaging System (Leica Biosystems).

### Immunohistochemistry

Neuroendocrine (chromogranin and synaptophysin) and MMR IHC was performed according to standard histological technique as previously described (Kushnir et al. 2014). Briefly, 5-µm thick sections from the FFPE tissue block were labeled using a Benchmark XT automated slide staining system (Ventana Medical Systems, Inc.) following standard protocols. Prediluted concentrations of the following monoclonal antibodies were used: anti-Chromogranin A (Ventana Medical Systems, Inc.; clone LK2H10; monoclonal); anti-Synaptophysin PMS2 (Cell Marque, Co.; clone MRQ-40; monoclonal); anti-MLH1 (Ventana Medical Systems, Inc.; clone M1; monoclonal), anti-PMS2 (Cell Marque, Co.; clone EPR3947; monoclonal), anti-MSH2 (Cell Marque, Co.; clone G219-1129; monoclonal), and anti-MSH6 (Ventana Medical Systems, Inc.; clone 44; monoclonal). Ventana's ultraView Universal DAB Detection Kit was utilized and staining-visualized using hydrogen peroxide substrate and a 3,3′-diaminodenzidine tetrahydrochloride (DAB) chromogen. Manufacture recommendations were adhered to for antigen retrieval conditions (Ventana CC1, EDTA-Tris, pH 8.0 solution). Nuclear staining for MLH1, PMS2, MSH2, and MSH6 was considered positive. Lymphocytes and uninvolved colonic mucosa were used as an internal positive control for the MMR markers. Normal control colon was used as a positive control for the chromogranin and synaptophysin IHC.

## ADDITIONAL INFORMATION

### Data Deposition and Access

Data were generated as part of routine patient care and consent was not obtained for data sharing in publicly accessible databases. The *FGFR2* amplification has been reported as a variant of unknown significance in ClinVar (http://www.ncbi.nlm.nih.gov/clinvar/) under accession number SCV000297799.1.

### Ethics Statement

These data were generated as part of routine patient care, have been reviewed by the Washington University Office of Human Research Protection, and do not meet the criteria for human studies research.

### Author Contributions

J.H.C, E.J.D., and C.E.C. contributed to study design. J.H.C, C.E.C, S.L., S.N.M., and E.J.D. contributed to the analysis and interpretation of genomic results. J.H.C. contributed to analysis and interpretation of the histologic results. K.A.V. contributed to figure design and layout. S.L. contributed to analysis and interpretation of FISH results. All authors contributed to manuscript writing, editing, and approval.

### Competing Interest Statement

C.E.C. and J.W.H. are consultants for PierianDx. E.J.D. is a consultant for Cofactor Genomics.

### Referees

Marilyn M. Li

Anonymous

## Supplementary Material

Supplemental Material
