# Supplementary material for: FGFR2 amplification in colorectal adenocarcinoma
Source: Cold Spring Harb Mol Case Stud. 2017 Nov;3(6):a001495. doi: 10.1101/mcs.a001495 (PMC5701301; doi:10.1101/mcs.a001495)
Supplement: Supplemental Material [file supp_3_6_a001495__index.html]

Supplemental Material 

# *FGFR2* amplification in colorectal adenocarcinoma

## Supplemental Material

- Supplemental\_Table\_S1.xlsx
- Supplemental\_Table\_S2\_QCmetrics.docx
