# Supplementary material for: FGFR2 amplification in colorectal adenocarcinoma
Source: Cold Spring Harb Mol Case Stud. 2017 Nov;3(6):a001495. doi: 10.1101/mcs.a001495 (PMC5701301; doi:10.1101/mcs.a001495)
Supplement: Supplemental Material [file supp_mcs.a001495_Supplemental_Table_S2_QCmetrics.docx]

| **Parameter** | **Patient sample** | **Reference average** |
| --- | --- | --- |
| Total reads | 20,094,270 | 14,851,242 |
| % mapped to genome | 94.80% | 94.0% |
| % mapped on target | 75.88% | 48.5% |
| On target reads | 14,455,272 | 6,622,724 |
| % of on target that are unique | 52.95% | 87.9% |
| Unique on target reads | 7,654,306 | 5,796,593 |
| Mean mapping quality | 69.99 | 69.98 |
| % positions ≤ 50x unique | 99.56% | 99.6% |
| % positions ≤ 400x unique | 97.41% | 93.4% |
| % positions ≤ 1000x unique | 35.27% | 59.2% |
| Average unique coverage (across full capture region) | 1560.0 | 1135 |

All SNV calls were reported only when depth of coverage >= 50x, and Fisher strand bias <= 75, and VAF > 0.1 OR VAF between 0.03-0.1 and strand bias not 0 or 1

All indel calls were reported only when depth >=50 and Fisher strand bias <= 75, and no adjacent homopolymer run longer than 7bp.
